# Attitudes to earlier advance care planning: a qualitative interview study in general practice

**DOI:** 10.3399/BJGP.2025.0393

**Published:** 2026-01-13

**Authors:** Sonya Bushell, Ishbel Winter-Luke, Elizabeth Dennis, Fliss EM Murtagh

**Affiliations:** 1 Wolfson Palliative Care Research Centre, Hull York Medical School, University of Hull, Hull, UK

**Keywords:** advance care planning, chronic disease, general practice, palliative medicine, public health, qualitative research

## Abstract

**Background:**

Advance care planning (ACP) research has often focused on those experiencing deteriorating health. There is limited research regarding attitudes to early ACP among people living with long-term conditions without advanced illness or severe frailty.

**Aim:**

To explore how people living with a non-cancer long-term condition understand ACP; and their preferences for how, if at all, these discussions should be undertaken, including within annual health reviews.

**Design and setting:**

Qualitative interviews undertaken with participants from general practices in village, market town, coastal town, and city settings within England, UK.

**Method:**

The interviews were semi-structured and in depth. The inclusion criteria were: >18 years old; registered at a participating practice; living with cardiovascular disease, diabetes mellitus, kidney disease, or chronic obstructive pulmonary disease. Thematic analysis used a critical realist approach. Participants contributed to a member checking process. This study had NHS ethics approval (23/PR/0078).

**Results:**

In total, 21 participants were recruited who were aged 61–91 years, eight were men, and 17 participants were living with multimorbidity. Participants discussed three forms of ACP: proactive planning, preparing for change, and discussing the end of life. Participants described early ACP as less distressing. Participants perceived ACP as an ongoing process, with early consultations encouraging discussion of existing preferences and preparing people for future decision making. Participants discussed how ACP could facilitate proactive and person-centred health care. Participants described the importance of normalising ACP.

**Conclusion:**

This study suggests that ACP may be well received and could be discussed earlier with adults living with long-term conditions, before onset of advanced illness or severe frailty.

## How this fits in

Advance care planning (ACP) may improve patient–physician communication, patient satisfaction, and the quality of end-of-life care. However, ACP is often only offered to those experiencing advanced illness or severe frailty. This qualitative research project identified an openness to early ACP among adults living with long-term conditions. Our study findings suggest that healthcare professionals could introduce ACP earlier to patients living with long-term conditions, and that there is potential to normalise ACP within healthcare and wider society.

## Introduction

Advance care planning (ACP) is *‘a process that supports adults at any age or stage of health in understanding and sharing their personal values, life goals, and preferences regarding future medical care’*.^1^ Despite this broad definition, ACP discussions are often reserved for those experiencing advanced illness or severe frailty.^2–4^ Evidence suggests ACP can improve patient–physician communication and caregivers’ understanding of patients’ preferences and may improve patient satisfaction and the quality of end-of-life care.^5–9^ However, ACP prevalence is low: for example, a same-day survey across 123 acute UK hospitals in 2018 found that only 4.8% of patients admitted under medical specialties had ACP documentation.^2^ Research shows that, although some patients wish to avoid or postpone ACP, others want to engage in early ACP.^4,10–13^

Patients with non-cancer conditions are less likely to receive ACP than those with malignancy.^14–16^ These patients are principally managed by primary care and could benefit from early ACP. Although there is increasing research on the use of ACP in primary care,^3,14^ such research has often focused on the use of ACP among people experiencing advanced illness or frailty. As research regarding the ReSPECT programme has shown, healthcare professionals may only discuss ACP after a diagnosis of a life-changing condition or after a deterioration in health.^3^ There has been limited research regarding the acceptability of ACP among those living with long-term conditions (LTCs) who were not at imminent risk of deteriorating health. The aim of this study was to explore how people living with a non-cancer LTC understood planning ahead discussions; to explore preferences for how, if at all, these discussions should be undertaken; and to explore concerns and expectations around including such discussions within annual health reviews.

## Method

This qualitative research project received NHS ethics approval (23/PR/0078). The study team comprised four clinical academics from a medicine background. The study team discussed study design, implementation, and findings within wider multidisciplinary research meetings to gain further clinical and researcher perspectives.

### Recruitment

Twenty general practices across an integrated care board in England, UK, were invited to participate. Of those, 10 practices participated in the project and five successfully recruited patients. Participants were recruited from practices in village, market town, coastal town, and suburban city settings.

Participating practices shared study information with potential participants via multiple mediums: waiting room materials, website and social media updates, texts to patients, verbal discussions during consultations, and emails to patient participation group members. Practices could select which method(s) to use in line with staff preferences and capabilities. Additionally, the study team shared information about the research project on Facebook™ within local community groups in line with ethics approval. The inclusion criteria for the study were: being >18 years old, registered at a participating practice, living with cardiovascular disease, diabetes mellitus, kidney disease, or chronic obstructive pulmonary disease. The exclusion criteria were: those who lack capacity to consent to research participation. The study was designed to capture the experiences of those living with both asymptomatic and symptomatic disease, and those experiencing frequent and less frequent monitoring in primary care.

### Data collection

Participants received an information sheet and consent form in advance of their interview. Participants could be interviewed face to face (at their local practice) or remotely (via telephone or Teams™). All participants chose face-to-face interviews. Participants were interviewed by the first author alone or, if desired, with a friend or family member (who could also participate in the interview). Written consent was gained from all participants (and those accompanying participants). Interviews were audio-recorded and transcribed verbatim. The first author produced field notes after each interview to provide contextual information and facilitate reflection. The first author was an early-career clinical academic with postgraduate training in qualitative research and clinical research ethics. Supervision was always available from the principal investigator.

Semi-structured, in-depth interviews took place between June 2023 and September 2024. The topic guide (Supplementary Information S1) was informed by the research questions and discussions with multidisciplinary clinicians and researchers, and was further refined during initial interviews in response to participants’ disclosures. Interviews were conducted in a flexible and supportive manner with open questions and active listening used to encourage extensive discussion.

Thematic analysis followed Braun and Clarke’s methodology^17^ and used a critical realist approach. Initial coding using NVivo (version 14) was conducted by the first author. Two members of the study team independently coded two randomly selected transcripts, and met the whole team to discuss discrepancies, to help develop and refine the coding tree. Throughout coding, the researcher kept a reflexive diary and participated in supervisory discussions with the principal investigator to reflect on and refine data analysis. The final thematic map was discussed among all members of the study team. To enhance the credibility of findings, the study team then wrote an interim report of the research findings and invited participants to give feedback during a member checking process.

## Results

Of the 26 people interested in research participation, three people did not meet the eligibility criteria and two people subsequently declined participation because of personal circumstances. In total, 21 participants were therefore interviewed (with two interviews including a relative or friend). Participants were aged between 61 and 91 years and included eight men and 13 women. Table 1 provides more demographic information. Significantly, participants lived in less deprived areas according to Index of Multiple Deprivation categorisation, and most were of White British ethnicity. Most participants were living with multiple LTCs rather than a single LTC. Most participants had low Rockwood Frailty Scores (<4), representing no frailty.

**Table 1.** Participants’ characteristics (*N* = 21)

Characteristics	Participants, *n*	Participants, %
**Age, years**		
<65	2	9.5
65–74	10	47.6
75–84	6	28.6
>84	3	14.3
**Rockwood Frailty Score**		
1–3 (fit/well/managing well)	14	66.7
4 (vulnerable)	4	19.1
5 (mild)	2	9.5
6 (moderate)	1	4.8
**Ethnicity**		
White British	16	76.2
White other	3	14.3
White and other ethnic backgrounds	1	4.8
Prefer not to say	1	4.8
**Major conditions**		
Hypertension	9	42.9
Atrial fibrillation	4	19.1
Coronary heart disease	5	23.8
Heart failure	1	4.8
Long-term respiratory disease (asthma, bronchiectasis, COPD)	5	23.8
Type 1 diabetes	1	4.8
Type 2 diabetes	3	14.3
Chronic kidney disease	2	9.5
Dementia (any type)	1	4.8
Cancer	1	4.8
**Multimorbidity**		
≥2 long-term conditions	17	81.0
≥3 long-term conditions	11	52.4
≥5 long-term conditions	3	14.3
**Index of Multiple Deprivation deciles^a^**		
5–6	8	38.1
7–8	5	23.8
9–10	7	33.3
Postcode not given	1	4.8

^a^The Index of Multiple Deprivation (IMD) is the official measure of relative deprivation in England (a decile of 1 represents the most deprived 10% of areas nationally and a decile of 10 represents the least deprived 10% of areas nationally). COPD = chronic obstructive pulmonary disease.

### Understanding of and engagement with ACP

The term ‘advance care planning’ was unfamiliar to most participants. Some understood ACP as a way of improving an individual’s health or improving health or care services:

*‘I thought I’m going to change the health service.’* (Participant 012, Male, 70s, Rockwood Frailty 1)

Others associated the term with end-of-life care, perhaps reflecting how ACP is presented within healthcare and society.

When asked about ‘planning ahead’, participants described multiple preparations. This included considering future living arrangements, making provisions for death (such as making a will), and completing lasting power of attorney (LPA) documentation. Many participants worried about social care and wanted to avoid care homes:

*‘We’re never going into a care home. That’s it!’* (Participant 008, Female, 70s, Rockwood Frailty 1)*.*

Although ACP has traditionally focused on medical decisions, participants discussed social, legal, and financial considerations (and, rarely, religious considerations) alongside and within medical decisions. We have reflected this broader approach to ACP throughout this article. Importantly, few participants had discussed the future with healthcare professionals or felt empowered to do so:

*‘There’s never really been an opportunity.’* (Participant 002, Male, 70s, Rockwood Frailty 1)

Some participants had engaged in ACP discussions in relation to a friend’s or relative’s health, although often only at the very end of life. Some participants described having to advocate for appropriate decision making at the end of life. Many participants had witnessed poor end-of-life experiences (before, during, and after the COVID-19 pandemic) that motivated them to engage in ACP. Participants were open to discussing ACP within health care. When discussing healthcare planning, participants often focused on end-of-life and emergency decisions. After discussing ACP in broad terms, participants were shown the ReSPECT (Recommended Summary Plan for Emergency Care and Treatment) form^18^ as an example of ACP used in the UK. Most participants perceived the form positively.

Participants described different types of ACP, and also discussed how the focus of ACP could vary depending on the context. The authors identified three different forms of ACP discussed by participants:

- proactive planning;
- preparing for change; and
- discussing end of life.

### Proactive planning

The first type of ACP refers to proactive planning. This involved making general preparations for the future while in reasonable health. This form of ACP was often conducted outside of healthcare and involved considering future living arrangements alongside medical decision making. In this context, ACP was seen as an administrative task rather than an emotional activity. Participants thought they would likely remain well for some time but wanted to be prepared anyway:

*‘* […] *but I think some people will always make future plans and it’s like having travel insurance.’* (Participant 011, Female, 70s, Rockwood Frailty 2)

Barriers to engaging in proactive planning included the belief that ACP was not yet necessary, a desire to focus on the present, a reluctance to consider future illness or death, and personal pressures such as being busy. Participants discussed how early decision making might be uninformed (such as nominating an LPA without discussing their healthcare preferences with the nominated person) and noted that people might change their decisions later. However, participants thought that it was easier to make healthcare decisions while experiencing good health:

‘*If you leave it until there is a real crisis situation* […] *feelings are heightened and everyone’s fearful, and you know you’re not necessarily going to be understanding what somebody in a calm, sensible state of mind would prefer.’* (Daughter of Participant 022 [Female, 90s, Rockwood Frailty 6])

### Preparing for change

The second understanding of ACP was preparing for change. Participants were aware that older people or people living with an LTC could experience worse health in future. Participants described the importance of offering ACP to people at risk of deteriorating health:

*‘I think it’s fair to ask people when they get older, as to what age I don't think it’s so much an age thing as a, a general health condition thing.’* (Participant 008, Female, 70s, Rockwood Frailty 1)

Participants were open to discussing ACP within LTC annual health reviews and consultations.

The degree to which participants experienced anxiety about their health related to personality traits, life circumstances, the extent to which they were symptomatic, their experiences of self-management and treatment burdens, and their understanding of risk. Participants wanted to receive information about their future health and wanted to discuss their treatment options:

*‘* […] *it’s like when you’re giving someone a new drug or something about it* [health condition] *or you’re going in for an operation? Yes, they have to talk about side effects or what may go wrong* […] I *think it’s nice to know what your options would be. Erm … yeah, your health care and your type of care that you can get.’* (Participant 007, Male, 60s, Rockwood Frailty 4)

Some participants felt anxious discussing the future. Other participants were comfortable discussing the future, particularly if they felt well supported or financially secure:

*‘My husband’s been recently diagnosed with a melanoma* […] *we could afford to either move or* […] *if we needed to get people in. So yeah, there is some planning in my head, but not to a great degree of worry basically.’* (Participant 011, Female, 70s, Rockwood Frailty 2)

### Discussing end of life

The third type of ACP refers to discussing the end of life. This involved explicit acknowledgement of, and planning for, an individual’s death. Some participants had only encountered this type of ACP and therefore associated ACP with end-of-life care. Because of this association, participants worried that discussing ACP with a patient could be misinterpreted as a sign that they were imminently dying:

*‘Unless, unless the person thinks when they see this* [ReSPECT form]*, my God, they think I’m gonna die, that, that could be the finishing touch of life!’* (Participant 009, Male, 70s, Rockwood Frailty 1)

Participants noted that ACP at any stage could involve thinking about death, but that death was more explicitly discussed with those experiencing advanced illness. Some participants were distressed by discussing the end of life:

*‘The terrifying thought can be a terrifying thought. Okay, so if it’s, it’s all done to help you.’* (Participant 010, Female, 60s, Rockwood Frailty 1)

Participants described how fear could limit some people’s engagement with ACP, particularly in the context of terminal illness:

*‘It’s probably a bit demoralising if you if, if you are suffering from well, it’s, especially if it’s an incurable disease.’* (Participant 012, Male, 70s, Rockwood Frailty 1)

However, other participants found it reassuring to discuss and plan their end-of-life care:

*‘I was very lucky, erm, some year, when I was eighty* […] *A nurse came, I was asked to come down and talk to a nurse* [about] *what I wanted for the future. And all this* [ReSPECT form] *was covered* […] *and I would like, you know, the end of life to be just peaceful and have my family with me.’* (Participant 003, Female, 80s, Rockwood Frailty 4)

Some participants described how an awareness of mortality helped them appreciate life more:

‘*I think there’s a sense in which it’s quite good to think about it* […] *you know, enjoying life, appreciating life, but being aware that it is a gift that, at some point we’ll die effectively.’* (Participant 018, Female, 60s, Rockwood Frailty 2)

Participants’ openness to discussing dying appeared to be influenced by previous experiences of end-of-life care and personality traits, rather than the nature of their health condition or frailty. However, most participants believed that it was less distressing to discuss dying while experiencing good health, as patients could be reassured that they were unlikely to die soon.

### Advance care planning across a lifetime

Most participants described early ACP as the start, rather than the completion, of an ongoing decision-making process. Participants described how ACP preferences could change in response to changing health or social circumstances. Therefore, some participants perceived very early discussion of ACP as unhelpful. Some participants spoke about the difficulty of timing ACP conversations:

*‘Not too late, not too early, it’s a bit like Goldilocks’ porridge.’* (Participant 010, Female, 60s, Rockwood Frailty 1)

Most participants valued early ACP providing they could revisit decisions in future. Some participants wanted to review ACP decisions at regular intervals, whereas others preferred to wait until their health changed. Some participants believed that their ACP preferences would remain constant, particularly regarding avoiding specific medical interventions.

**Figure 1.**
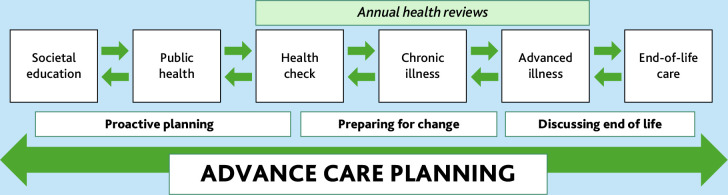
Advance care planning across a lifetime.

Informed by the interview findings, we present a conceptual framework (Figure 1) for how ACP could be employed across a lifetime. Early ACP (proactive planning) could occur in non-healthcare contexts and would involve planning for living alongside planning for dying. Later ACP would focus more on healthcare decisions and end-of-life care. Generally, participants described earlier ACP conversations as less emotionally taxing:

*‘It’s better to talk to people like me who are still feeling healthy* […] *because I’m not thinking you’re saying this to me because I'm dying basically.’* (Participant 011, Female, 70s, Rockwood Frailty 2)

Additionally, participants described how early conversations could normalise ACP and prepare people for future decision making:

*‘If it was there from the start, it would be less of an issue for the healthcare professionals having to mention it.’* (Participant 006, Female, 70s, Rockwood Frailty 3)

### Implementing ACP in primary care

Participants discussed how ACP could be integrated into consultations and annual health reviews in primary care. Most participants were open to discussing ACP during their health reviews and described how this could normalise ACP:

*‘Just try and make it a normal part of healthcare.’* (Participant 008, Female, 70s, Rockwood Frailty 1)

Participants described how annual health reviews could enable regular ACP reviews. Most participants received annual health reviews. Most perceived these reviews as reassuring and essential, although some described them as anxiety inducing, unhelpful, or impersonal.

Most participants trusted nurses to discuss and document ACP, with some trusting healthcare assistants. Some participants preferred nurses to doctors because of better rapport and longer appointments. Other participants, particularly those with complex conditions, wanted to discuss ACP with doctors who could provide detailed information and guidance. These participants described ACP as requiring clinical decision making and explanations beyond the automatic documentation of patients’ wishes.

Participants perceived primary care as less accessible, and less person-centred, since the COVID-19 pandemic. Most, although not all, participants valued continuity of care. Participants described how continuity of care could build trust, encourage honesty, and support personalised decision making:

*‘* […] *and it takes time to build up trust* […] *because people don’t kind of know the background, they might not be giving you appropriate advice.’* (Participant 018, Female, 60s, Rockwood Frailty 2)

### ACP and proactive health care

Participants wanted to receive more information around managing their health now and in the future:

*‘I said is there, is there* [laughs] *an NHS pamphlet for ageing?’* (Participant 002, Male, 70s, Rockwood Frailty 1)

Annual health reviews rarely covered such topics. There was strong desire for more proactive health care:

*‘Try and be more proactive with people before things get worse and you spend a fortune putting people right.’* (Participant 007, Male, 60s, Rockwood Frailty 4)

Participants discussed many ideas for more proactive health care including better preventative health care, information on self-management, improved communication within and between health and social care systems, more person-centred care, and continuity of care. Participants believed that ACP could support proactive care through improving communication between patients and professionals:

*‘But it was just stating what her wishes were, were, like what mattered to her when she was in hospital, about what treatment, what care and help she needed.’* (Participant 013, Female, 60s, Rockwood Frailty 2)

Additionally, some participants described how ACP could be integrated into preventative healthcare interventions such as screening programmes:

*‘Once you get to sixty, you get certain things sent out in post and I think why can't this be included in a check like that then’* (Participant 007, Male, 60s, Rockwood Frailty 4)

Participants valued ACP when it was perceived as facilitating more proactive health care rather than solely planning for end-of-life care.

### Promoting ACP

Most participants felt that ACP should be more widely discussed:

*‘I, I think people should, I think people should do it routinely.’* (Participant 001, Male, 70s, Rockwood Frailty 4)

Participants discussed challenging taboos around illness and death:

*‘People just don’t discuss it even down to their wills, funerals* […] *Erm but logically you should do.’* (Participant 007, Male, 60s, Rockwood Frailty 4)

This was particularly emphasised during the member checking process.

Participants suggested that practices and other healthcare institutions could share more information about ACP, either directly (via healthcare consultations or personal letters) or indirectly (via posters, leaflets, websites, social media, or generic mass messaging). Some participants wanted ACP information sent to everyone, whereas others suggested targeting those over a certain age or living with certain conditions. Participants recognised that some people might not want to consider ACP but did not want this to limit information sharing. Participants discussed the importance of trust and sensitivity when sharing information with people:

‘*If it comes in, in a caring way, I think that’s, that would be fine.’* (Participant 004, Female, 80s, Rockwood Frailty 5)

Many participants also discussed the role of community centres, workplaces, schools, and festivals in promoting ACP:

*‘But even if surgeries or, or local community centres even, could hold things that feel informal and unpressured like coffee mornings when people could come in and just discuss the whole gamut of things’* (Participant 017, Female, 60s, Rockwood Frailty 4)

Participants discussed how solicitors and registrars could signpost to ACP before life events such as marriage, buying a first home, or retirement. Participants also discussed the need to remove financial barriers to ACP, such as reducing the cost of registering an LPA.

Finally, participants discussed using print, broadcasting, and online media to promote awareness of ACP. Participants spoke about engaging the public through example scenarios, personal testimonials, or fictional stories. Participants discussed how celebrities could promote behaviour change:

*‘But I think if you have an athlete* […] “*I’m thinking of the long term and my family, and I’ve done a will and a lasting power of attorney and one of these with my doctor.” I think that’s the sort of thing you would need to get people thinking, oh well, they’re doing it.’* (Participant 011, Female, 70s, Rockwood Frailty 2)

## Discussion

### Summary

In this novel study, we explored attitudes to ACP among people living with non-cancer LTCs. Significantly, we found that participants were open to starting ACP discussions earlier. Historically ACP has been associated with advanced illness or end-of-life care. This study suggests that ACP should instead be understood as a broad term encompassing different conversations in different contexts (proactive planning, preparing for change, and facing mortality). Participants described how discussing ACP early could reduce distress around decision making and normalise ACP. Additionally, ACP could encourage more proactive health care by facilitating communication and person-centred care.

Participants suggested that ACP could be incorporated into preventative healthcare (such as annual health reviews or screening programmes). Participants also suggested ways to advertise ACP via healthcare institutions, community centres (including schools and registry offices), and national campaigns.

### Strengths and limitations

This study focused on an underresearched area by focusing on a population not already experiencing advanced illness or severe frailty. There was diversity in the study population regarding participants’ conditions and a reasonably broad age range. However, most participants were White British, all lived in less deprived areas, and there was an absence of people under 60 years. People with such characteristics may have fewer competing demands on their time, have greater trust in healthcare systems, and experience fewer barriers to accessing health care and research.^19^ Our non-diverse study population unfortunately limits the transferability of study findings, as previous research has identified additional barriers to ACP among ethnic minority and socioeconomically deprived populations.^20,21^

This was a small study; recruitment was challenging owing to primary care pressures and a reliance on the interest and capabilities of practice teams. As a result of the diversity in individuals’ medical conditions and experiences, new insights would likely have been recorded if recruitment had continued. However, data saturation was achieved around the core themes, including the different understandings of ACP and promoting ACP within health care and the wider society.

The first author’s use of open questioning and rapport-building during interviews facilitated in-depth rich findings. The first author occupied an insider–outsider status as an early-career clinician, carrying an understanding of medical practice without any firm loyalties to institutions or specific ways of working.

This study may have been affected by self-selection bias as those who participated were likely more comfortable discussing their future health care. However, many participants had limited understanding of ACP prior to study participation, and both positive and negative attitudes to ACP were encountered. Moreover, participants with positive attitudes to ACP were asked to reflect on the perspectives of others who might not engage in ACP.

Significantly, this was a snapshot study rather than a longitudinal one. There was no follow-up regarding participants’ future engagement with ACP (and any effects on healthcare outcomes). People may respond differently to ACP during a brief healthcare appointment rather than an extended conversation. There was no follow-up regarding consistency of participants’ attitudes and decisions over time and with changing illness.

This study project was guided by a critical realist approach suited to qualitative healthcare research. The first author’s use of a reflexive diary and regular supervisory meetings facilitated reflection on how their personal and professional background could influence study findings. Members of the study team independently coded two random transcripts to increase rigour. A member checking process and regular discussion with the study team was used to reduce lone researcher bias and increase the credibility of findings.

### Comparison with existing literature

Our study identified that participants had thought about the future but had not discussed this with healthcare professionals. Other studies have reported similar findings and identified various barriers and facilitators to ACP discussions.^13,22–24^ Some have suggested that reframing ACP within ‘future care planning’ could encourage patients and clinicians to prepare for, and adapt to, future changes in health or care needs.^25,26^ Others have discussed promoting ACP in non-medical contexts to increase awareness.^27,28^ Participants in our study demonstrated positive attitudes towards information sharing about ACP in non-medical contexts.

Systematic reviews have identified varying attitudes to ACP among patients, carers, and members of the public.^4,21,29^ The literature presents recurring concerns around ACP including poor understanding or trust regarding ACP (sometimes relating to personal or cultural experiences of health services), finding discussions distressing, and feeling that ACP is not yet necessary.^4,10,21,30–32^ Although participants in our study shared some of these concerns, most held positive attitudes to discussing ACP. This suggests that there may be a favourable window to start conversations about ACP among older adults living with chronic disease. Notably, other studies within this population have found positive attitudes towards ACP.^33,34^ Other studies among older adults have identified more mixed attitudes to ACP, although these included apparently frailer study populations.^31,32,35^ Previous studies have explored how ACP involves contemplating mortality and may cause distress.^35,36^ Participants in this study described early ACP as less distressing as death was not imminent and early ACP involved planning for living alongside planning for dying.

Participants discussed how ACP preferences could change as an individual’s health or social circumstances changed. The literature suggests that some patients’ ACP preferences change with time^35,37–39^ and that goal-concordant care is not always achieved.^5,40^ One interpretation of this evidence is that ACP is unhelpful.^41,42^ Another interpretation is that care planning remains beneficial but should be understood as an ongoing decision-making and information-sharing process.^43,44^ Participants in this study discussed how introducing ACP early could facilitate person-centred care in line with existing preferences, reduce stigma around taboo topics, and prepare people for future decision making.

There is increasing discussion about how best to improve health care. Our study participants wanted more proactive health care and thought that ACP could support this. Participants expressed diverse views on how best to improve health care, from system-level preventative interventions to better-quality patient–professional relationships. This reflects current debates within health care as to the meaning of general practice and where best to focus professionals’ time.^33^ Primary care teams are experiencing significant challenges regarding workloads and continuity of care, so it is reasonable to consider the appropriateness of directing primary care resources towards ACP for people experiencing relatively stable health. There is mixed evidence as to whether ACP improves healthcare system outcomes.^9,40,45^ However, ACP may improve communication and connection, support acceptance, and aid person-centred care.^3,9,32,36^ ACP, if done well, could improve patient and professional satisfaction in general practice.

### Implications for research and practice

Our study findings demonstrate that healthcare professionals could discuss ACP earlier with patients, and that such discussions might be valued and less distressing than those conducted during advanced illness. Future research should explore whether these findings are transferable to other populations, including inner-city and ethnic minority populations, as previous studies have identified barriers to ACP among these populations.^21^ Given primary care pressures, there is a need to understand clinicians’ perspectives on early ACP and assess the resources needed to train and support staff delivering ACP. Future research could evaluate the feasibility and acceptability of early ACP interventions for those with LTCs in primary care. Such research could explore which healthcare professionals are best placed to deliver early ACP interventions, how best to train and support staff delivering ACP, and the effect of early ACP interventions on health outcomes and continuity of care.

Additionally, our study findings suggest that public health campaigns and societal institutions could be used to promote awareness of ACP. Future research could explore how best to share information about ACP with the public and the feasibility and acceptability of incorporating ACP within public health interventions.
